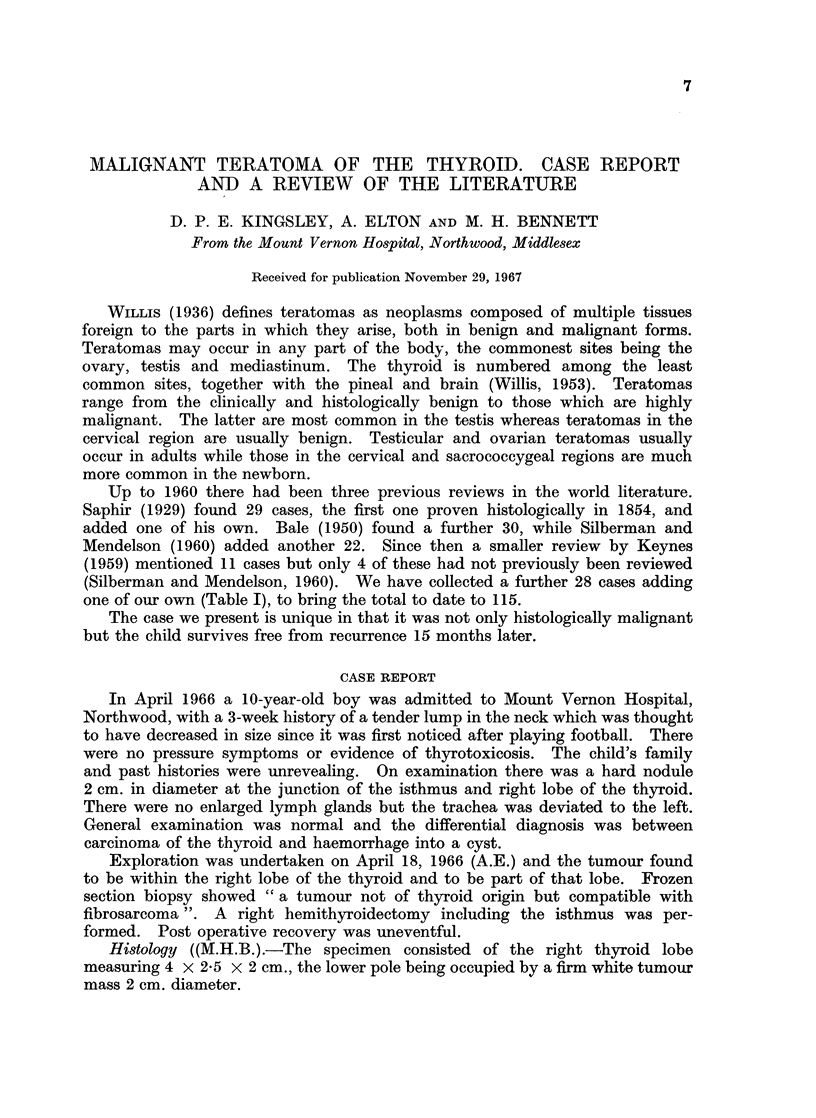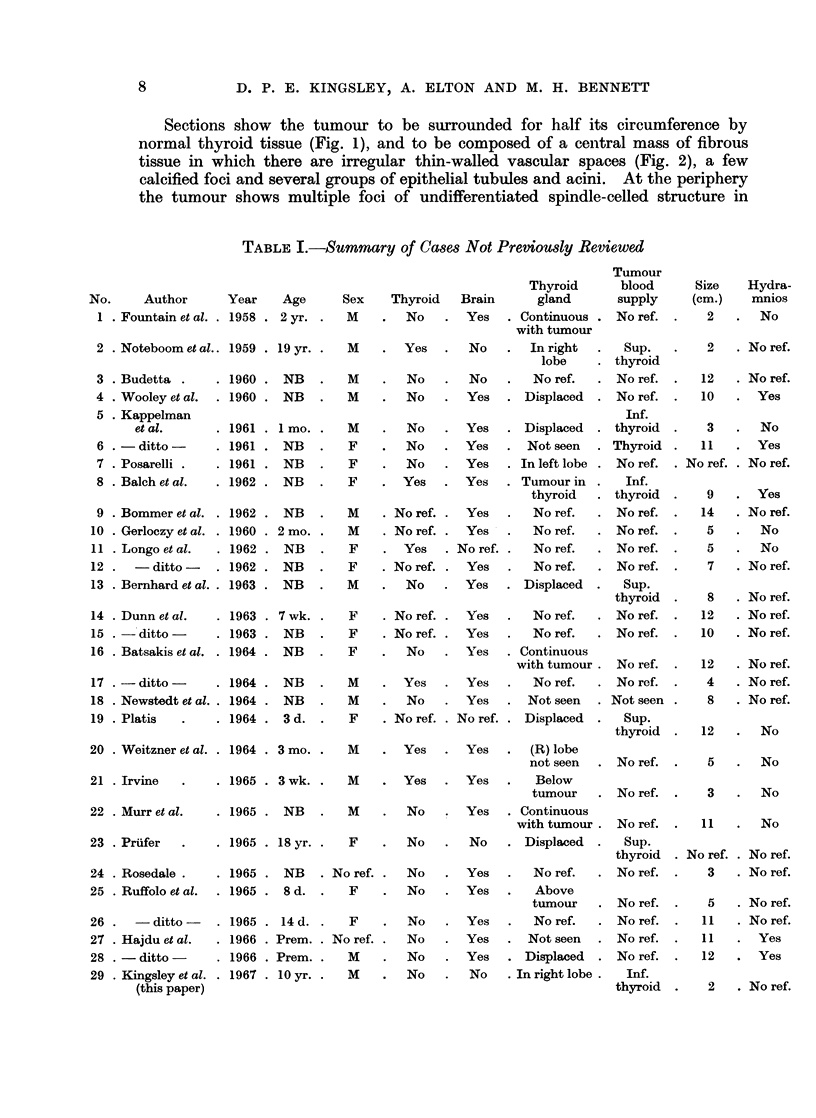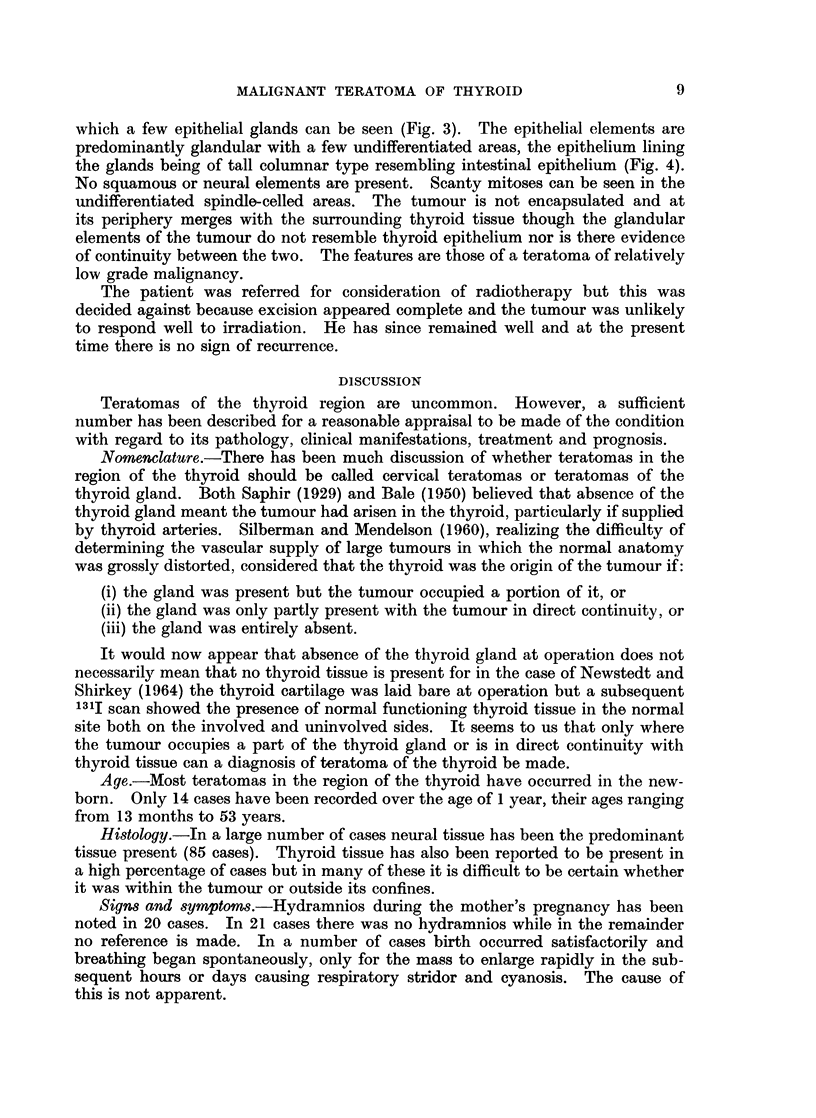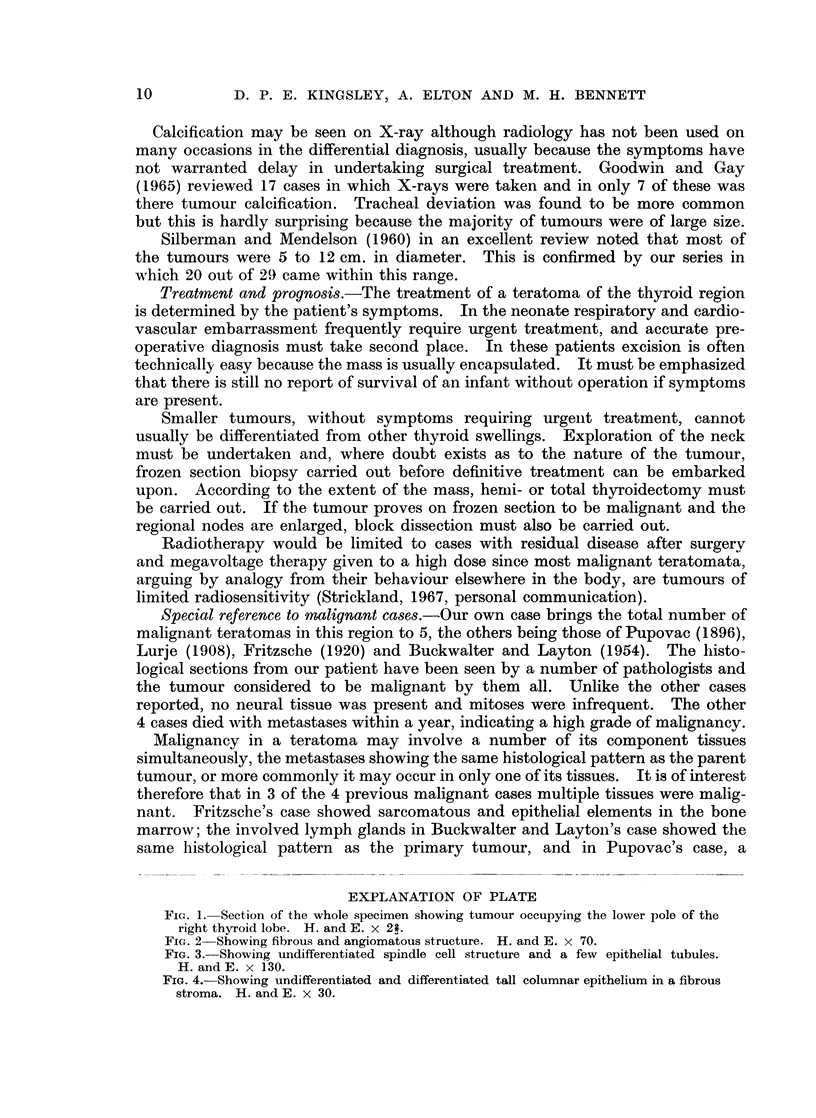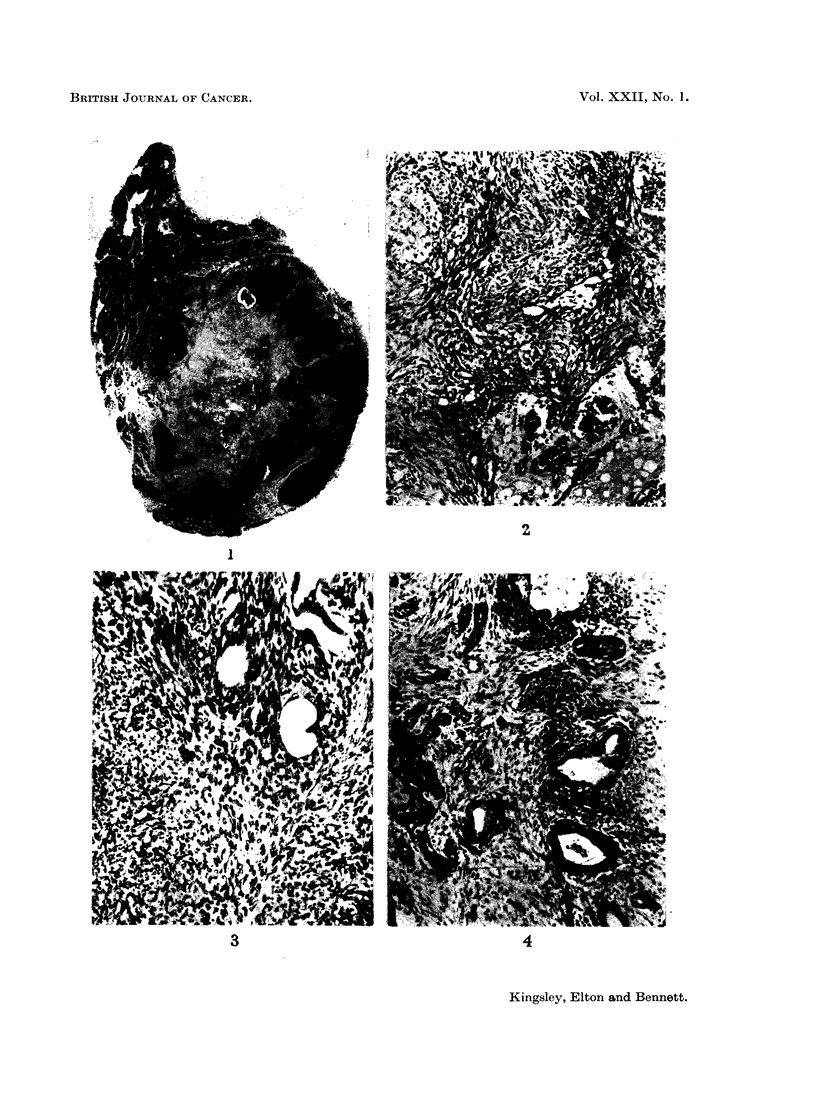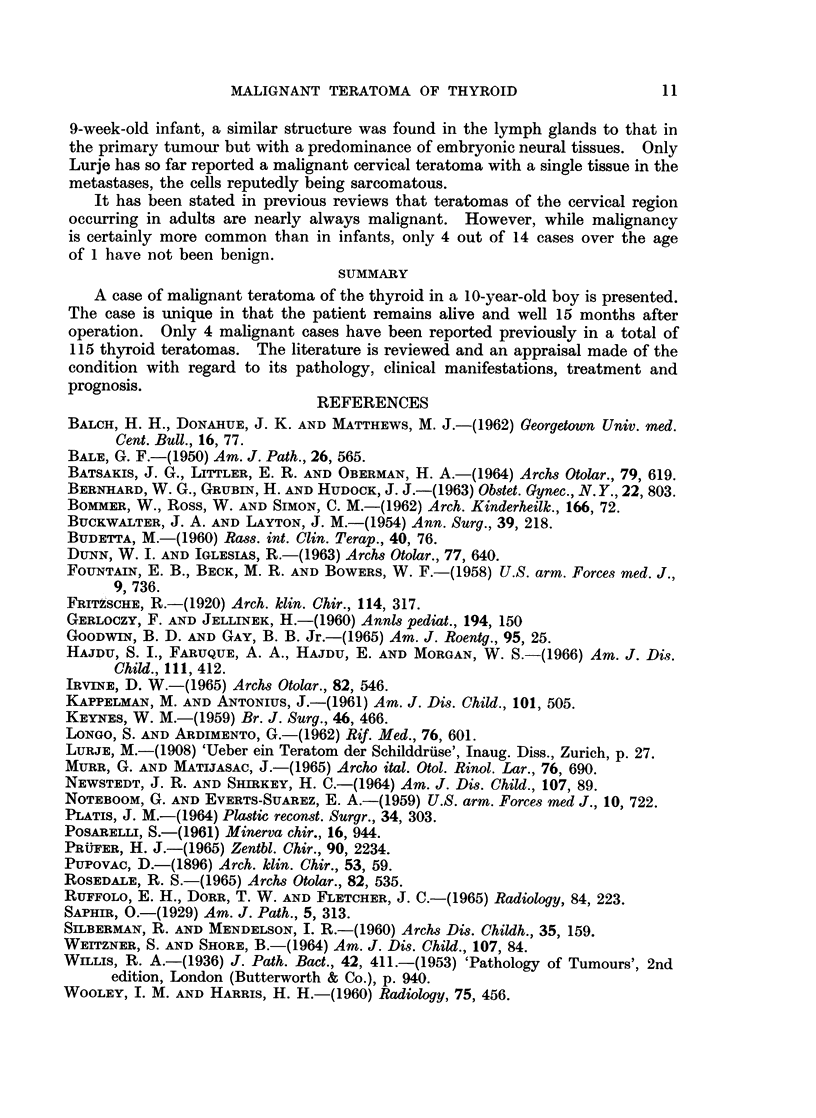# Malignant Teratoma of the Thyroid. Case Report and a Review of the Literature

**Published:** 1968-03

**Authors:** D. P. E. Kingsley, A. Elton, M. H. Bennett

## Abstract

**Images:**


### MALIGNANT TERATOMA OF THE THYROID. CASE REPORT AND A REVIEW OF THE LITERATURE

D. P. E. KINGSLEY, A. ELTON AND M. H. BENNETT

*From the Mount Vernon Hospital, Northwood, Middlesex*

Received for publication November 29, 1967

WILLIS (1936) defines teratomas as neoplasms composed of multiple tissues foreign to the parts in which they arise, both in benign and malignant forms. Teratomas may occur in any part of the body, the commonest sites being the ovary, testis and mediastinum. The thyroid is numbered among the least common sites, together with the pineal and brain (Willis, 1953). Teratomas range from the clinically and histologically benign to those which are highly malignant. The latter are most common in the testis whereas teratomas in the cervical region are usually benign. Testicular and ovarian teratomas usually occur in adults while those in the cervical and sacrococcygeal regions are much more common in the newborn.

Up to 1960 there had been three previous reviews in the world literature. Saphir (1929) found 29 cases, the first one proven histologically in 1854, and added one of his own. Bale (1950) found a further 30, while Silberman and Mendelson (1960) added another 22. Since then a smaller review by Keynes (1959) mentioned 11 cases but only 4 of these had not previously been reviewed (Silberman and Mendelson, 1960). We have collected a further 28 cases adding one of our own (Table I), to bring the total to date to 115.

The case we present is unique in that it was not only histologically malignant but the child survives free from recurrence 15 months later.

### CASE REPORT

In April 1966 a 10-year-old boy was admitted to Mount Vernon Hospital, Northwood, with a 3-week history of a tender lump in the neck which was thought to have decreased in size since it was first noticed after playing football. There were no pressure symptoms or evidence of thyrotoxicosis. The child's family and past histories were unrevealing. On examination there was a hard nodule 2 cm. in diameter at the junction of the isthmus and right lobe of the thyroid. There were no enlarged lymph glands but the trachea was deviated to the left. General examination was normal and the differential diagnosis was between carcinoma of the thyroid and haemorrhage into a cyst.

Exploration was undertaken on April 18, 1966 (A.E.) and the tumour found to be within the right lobe of the thyroid and to be part of that lobe. Frozen section biopsy showed " a tumour not of thyroid origin but compatible with fibrosarcoma ". A right hemithyroidectomy including the isthmus was performed. Post operative recovery was uneventful.

*Histology* ((M.H.B.).—The specimen consisted of the right thyroid lobe measuring 4 × 2·5 × 2 cm., the lower pole being occupied by a firm white tumour mass 2 cm. diameter.

Sections show the tumour to be surrounded for half its circumference by normal thyroid tissue (Fig. 1), and to be composed of a central mass of fibrous tissue in which there are irregular thin-walled vascular spaces (Fig. 2), a few calcified foci and several groups of epithelial tubules and acini. At the periphery the tumour shows multiple foci of undifferentiated spindle-celled structure in

TABLE I.—*Summary of Cases Not Previously Reviewed*

| No. | Author | Year | Age | Sex | Thyroid | Brain | Thyroid gland | Tumour blood supply | Size (cm.) | Hydramnios |
|---|---|---|---|---|---|---|---|---|---|---|
| 1 | Fountain *et al.* | 1958 | 2 yr. | M | No | Yes | Continuous with tumour | No ref. | 2 | No |
| 2 | Noteboom *et al.* | 1959 | 19 yr. | M | Yes | No | In right lobe | Sup. thyroid | 2 | No ref. |
| 3 | Budetta | 1960 | NB | M | No | No | No ref. | No ref. | 12 | No ref. |
| 4 | Wooley *et al.* | 1960 | NB | M | No | Yes | Displaced | No ref. | 10 | Yes |
| 5 | Kappelman *et al.* | 1961 | 1 mo. | M | No | Yes | Displaced | Inf. thyroid | 3 | No |
| 6 | — ditto — | 1961 | NB | F | No | Yes | Not seen | Thyroid | 11 | Yes |
| 7 | Posarelli | 1961 | NB | F | No | Yes | In left lobe | No ref. | No ref. | No ref. |
| 8 | Balch *et al.* | 1962 | NB | F | Yes | Yes | Tumour in thyroid | Inf. thyroid | 9 | Yes |
| 9 | Bommer *et al.* | 1962 | NB | M | No ref. | Yes | No ref. | No ref. | 14 | No ref. |
| 10 | Gerloczy *et al.* | 1960 | 2 mo. | M | No ref. | Yes | No ref. | No ref. | 5 | No |
| 11 | Longo *et al.* | 1962 | NB | F | Yes | No ref. | No ref. | No ref. | 5 | No |
| 12 | — ditto — | 1962 | NB | F | No ref. | Yes | No ref. | No ref. | 7 | No ref. |
| 13 | Bernhard *et al.* | 1963 | NB | M | No | Yes | Displaced | Sup. thyroid | 8 | No ref. |
| 14 | Dunn *et al.* | 1963 | 7 wk. | F | No ref. | Yes | No ref. | No ref. | 12 | No ref. |
| 15 | — ditto — | 1963 | NB | F | No ref. | Yes | No ref. | No ref. | 10 | No ref. |
| 16 | Batsakis *et al.* | 1964 | NB | F | No | Yes | Continuous with tumour | No ref. | 12 | No ref. |
| 17 | — ditto — | 1964 | NB | M | Yes | Yes | No ref. | No ref. | 4 | No ref. |
| 18 | Newstedt *et al.* | 1964 | NB | M | No | Yes | Not seen | Not seen | 8 | No ref. |
| 19 | Platis | 1964 | 3 d. | F | No ref. | No ref. | Displaced | Sup. thyroid | 12 | No |
| 20 | Weitzner *et al.* | 1964 | 3 mo. | M | Yes | Yes | (R) lobe not seen | No ref. | 5 | No |
| 21 | Irvine | 1965 | 3 wk. | M | Yes | Yes | Below tumour | No ref. | 3 | No |
| 22 | Murr *et al.* | 1965 | NB | M | No | Yes | Continuous with tumour | No ref. | 11 | No |
| 23 | Prüfer | 1965 | 18 yr. | F | No | No | Displaced | Sup. thyroid | No ref. | No ref. |
| 24 | Rosedale | 1965 | NB | No ref. | No | Yes | No ref. | No ref. | 3 | No ref. |
| 25 | Ruffolo *et al.* | 1965 | 8 d. | F | No | Yes | Above tumour | No ref. | 5 | No ref. |
| 26 | — ditto — | 1965 | 14 d. | F | No | Yes | No ref. | No ref. | 11 | No ref. |
| 27 | Hajdu *et al.* | 1966 | Prem. | No ref. | No | Yes | Not seen | No ref. | 11 | Yes |
| 28 | — ditto — | 1966 | Prem. | M | No | Yes | Displaced | No ref. | 12 | Yes |
| 29 | Kingsley *et al.* (this paper) | 1967 | 10 yr. | M | No | No | In right lobe | Inf. thyroid | 2 | No ref. |

which a few epithelial glands can be seen (Fig. 3). The epithelial elements are predominantly glandular with a few undifferentiated areas, the epithelium lining the glands being of tall columnar type resembling intestinal epithelium (Fig. 4). No squamous or neural elements are present. Scanty mitoses can be seen in the undifferentiated spindle-celled areas. The tumour is not encapsulated and at its periphery merges with the surrounding thyroid tissue though the glandular elements of the tumour do not resemble thyroid epithelium nor is there evidence of continuity between the two. The features are those of a teratoma of relatively low grade malignancy.

The patient was referred for consideration of radiotherapy but this was decided against because excision appeared complete and the tumour was unlikely to respond well to irradiation. He has since remained well and at the present time there is no sign of recurrence.

### DISCUSSION

Teratomas of the thyroid region are uncommon. However, a sufficient number has been described for a reasonable appraisal to be made of the condition with regard to its pathology, clinical manifestations, treatment and prognosis.

*Nomenclature.*—There has been much discussion of whether teratomas in the region of the thyroid should be called cervical teratomas or teratomas of the thyroid gland. Both Saphir (1929) and Bale (1950) believed that absence of the thyroid gland meant the tumour had arisen in the thyroid, particularly if supplied by thyroid arteries. Silberman and Mendelson (1960), realizing the difficulty of determining the vascular supply of large tumours in which the normal anatomy was grossly distorted, considered that the thyroid was the origin of the tumour if:

(i) the gland was present but the tumour occupied a portion of it, or
(ii) the gland was only partly present with the tumour in direct continuity, or
(iii) the gland was entirely absent.

It would now appear that absence of the thyroid gland at operation does not necessarily mean that no thyroid tissue is present for in the case of Newstedt and Shirkey (1964) the thyroid cartilage was laid bare at operation but a subsequent $^{131}I$ scan showed the presence of normal functioning thyroid tissue in the normal site both on the involved and uninvolved sides. It seems to us that only where the tumour occupies a part of the thyroid gland or is in direct continuity with thyroid tissue can a diagnosis of teratoma of the thyroid be made.

*Age.*—Most teratomas in the region of the thyroid have occurred in the newborn. Only 14 cases have been recorded over the age of 1 year, their ages ranging from 13 months to 53 years.

*Histology.*—In a large number of cases neural tissue has been the predominant tissue present (85 cases). Thyroid tissue has also been reported to be present in a high percentage of cases but in many of these it is difficult to be certain whether it was within the tumour or outside its confines.

*Signs and symptoms.*—Hydramnios during the mother's pregnancy has been noted in 20 cases. In 21 cases there was no hydramnios while in the remainder no reference is made. In a number of cases birth occurred satisfactorily and breathing began spontaneously, only for the mass to enlarge rapidly in the subsequent hours or days causing respiratory stridor and cyanosis. The cause of this is not apparent.

Calcification may be seen on X-ray although radiology has not been used on many occasions in the differential diagnosis, usually because the symptoms have not warranted delay in undertaking surgical treatment. Goodwin and Gay (1965) reviewed 17 cases in which X-rays were taken and in only 7 of these was there tumour calcification. Tracheal deviation was found to be more common but this is hardly surprising because the majority of tumours were of large size.

Silberman and Mendelson (1960) in an excellent review noted that most of the tumours were 5 to 12 cm. in diameter. This is confirmed by our series in which 20 out of 29 came within this range.

*Treatment and prognosis.*—The treatment of a teratoma of the thyroid region is determined by the patient's symptoms. In the neonate respiratory and cardiovascular embarrassment frequently require urgent treatment, and accurate preoperative diagnosis must take second place. In these patients excision is often technically easy because the mass is usually encapsulated. It must be emphasized that there is still no report of survival of an infant without operation if symptoms are present.

Smaller tumours, without symptoms requiring urgent treatment, cannot usually be differentiated from other thyroid swellings. Exploration of the neck must be undertaken and, where doubt exists as to the nature of the tumour, frozen section biopsy carried out before definitive treatment can be embarked upon. According to the extent of the mass, hemi- or total thyroidectomy must be carried out. If the tumour proves on frozen section to be malignant and the regional nodes are enlarged, block dissection must also be carried out.

Radiotherapy would be limited to cases with residual disease after surgery and megavoltage therapy given to a high dose since most malignant teratomata, arguing by analogy from their behaviour elsewhere in the body, are tumours of limited radiosensitivity (Strickland, 1967, personal communication).

*Special reference to malignant cases.*—Our own case brings the total number of malignant teratomas in this region to 5, the others being those of Pupovac (1896), Lurje (1908), Fritzsche (1920) and Buckwalter and Layton (1954). The histological sections from our patient have been seen by a number of pathologists and the tumour considered to be malignant by them all. Unlike the other cases reported, no neural tissue was present and mitoses were infrequent. The other 4 cases died with metastases within a year, indicating a high grade of malignancy.

Malignancy in a teratoma may involve a number of its component tissues simultaneously, the metastases showing the same histological pattern as the parent tumour, or more commonly it may occur in only one of its tissues. It is of interest therefore that in 3 of the 4 previous malignant cases multiple tissues were malignant. Fritzsche's case showed sarcomatous and epithelial elements in the bone marrow; the involved lymph glands in Buckwalter and Layton's case showed the same histological pattern as the primary tumour, and in Pupovac's case, a

---

EXPLANATION OF PLATE

FIG. 1.—Section of the whole specimen showing tumour occupying the lower pole of the right thyroid lobe. H. and E. × 2$\frac{2}{3}$.

FIG. 2—Showing fibrous and angiomatous structure. H. and E. × 70.

FIG. 3.—Showing undifferentiated spindle cell structure and a few epithelial tubules. H. and E. × 130.

FIG. 4.—Showing undifferentiated and differentiated tall columnar epithelium in a fibrous stroma. H. and E. × 30.

1

2

3

4

9-week-old infant, a similar structure was found in the lymph glands to that in the primary tumour but with a predominance of embryonic neural tissues. Only Lurje has so far reported a malignant cervical teratoma with a single tissue in the metastases, the cells reputedly being sarcomatous.

It has been stated in previous reviews that teratomas of the cervical region occurring in adults are nearly always malignant. However, while malignancy is certainly more common than in infants, only 4 out of 14 cases over the age of 1 have not been benign.

SUMMARY

A case of malignant teratoma of the thyroid in a 10-year-old boy is presented. The case is unique in that the patient remains alive and well 15 months after operation. Only 4 malignant cases have been reported previously in a total of 115 thyroid teratomas. The literature is reviewed and an appraisal made of the condition with regard to its pathology, clinical manifestations, treatment and prognosis.

### REFERENCES

Balch, H. H., Donahue, J. K. and Matthews, M. J.—(1962) *Georgetown Univ. med. Cent. Bull.*, **16**, 77.
Bale, G. F.—(1950) *Am. J. Path.*, **26**, 565.
Batsakis, J. G., Littler, E. R. and Oberman, H. A.—(1964) *Archs Otolar.*, **79**, 619.
Bernhard, W. G., Grubin, H. and Hudock, J. J.—(1963) *Obstet. Gynec., N.Y.*, **22**, 803.
Bommer, W., Ross, W. and Simon, C. M.—(1962) *Arch. Kinderheilk.*, **166**, 72.
Buckwalter, J. A. and Layton, J. M.—(1954) *Ann. Surg.*, **39**, 218.
Budetta, M.—(1960) *Rass. int. Clin. Terap.*, **40**, 76.
Dunn, W. I. and Iglesias, R.—(1963) *Archs Otolar.*, **77**, 640.
Fountain, E. B., Beck, M. R. and Bowers, W. F.—(1958) *U.S. arm. Forces med. J.*, **9**, 736.
Fritzsche, R.—(1920) *Arch. klin. Chir.*, **114**, 317.
Gerloczy, F. and Jellinek, H.—(1960) *Annls pediat.*, **194**, 150
Goodwin, B. D. and Gay, B. B. Jr.—(1965) *Am. J. Roentg.*, **95**, 25.
Hajdu, S. I., Faruque, A. A., Hajdu, E. and Morgan, W. S.—(1966) *Am. J. Dis. Child.*, **111**, 412.
Irvine, D. W.—(1965) *Archs Otolar.*, **82**, 546.
Kappelman, M. and Antonius, J.—(1961) *Am. J. Dis. Child.*, **101**, 505.
Keynes, W. M.—(1959) *Br. J. Surg.*, **46**, 466.
Longo, S. and Ardimento, G.—(1962) *Rif. Med.*, **76**, 601.
Lurje, M.—(1908) 'Ueber ein Teratom der Schilddrüse', Inaug. Diss., Zurich, p. 27.
Murr, G. and Matijasac, J.—(1965) *Archo ital. Otol. Rinol. Lar.*, **76**, 690.
Newstedt, J. R. and Shirkey, H. C.—(1964) *Am. J. Dis. Child.*, **107**, 89.
Noteboom, G. and Everts-Suarez, E. A.—(1959) *U.S. arm. Forces med J.*, **10**, 722.
Platis, J. M.—(1964) *Plastic reconst. Surgr.*, **34**, 303.
Posarelli, S.—(1961) *Minerva chir.*, **16**, 944.
Prüfer, H. J.—(1965) *Zentbl. Chir.*, **90**, 2234.
Pupovac, D.—(1896) *Arch. klin. Chir.*, **53**, 59.
Rosedale, R. S.—(1965) *Archs Otolar.*, **82**, 535.
Ruffolo, E. H., Dorr, T. W. and Fletcher, J. C.—(1965) *Radiology*, 84, 223.
Saphir, O.—(1929) *Am. J. Path.*, **5**, 313.
Silberman, R. and Mendelson, I. R.—(1960) *Archs Dis. Childh.*, **35**, 159.
Weitzner, S. and Shore, B.—(1964) *Am. J. Dis. Child.*, **107**, 84.
Willis, R. A.—(1936) *J. Path. Bact.*, **42**, 411.—(1953) 'Pathology of Tumours', 2nd edition, London (Butterworth & Co.), p. 940.
Wooley, I. M. and Harris, H. H.—(1960) *Radiology*, **75**, 456.